# Differential effects of 11 years of long-term injectable testosterone undecanoate therapy on anthropometric and metabolic parameters in hypogonadal men with normal weight, overweight and obesity in comparison with untreated controls: real-world data from a controlled registry study

**DOI:** 10.1038/s41366-019-0517-7

**Published:** 2020-01-28

**Authors:** F. Saad, G. Doros, K. S. Haider, A. Haider

**Affiliations:** 1 Medical Affairs Andrology, Bayer AG, Berlin, Germany; 2 Gulf Medical University School of Medicine, Ajman, UAE; 3 Department of Epidemiology and Statistics, Boston University School of Public Health, Boston, MA USA; 4 Private Urology Practice, Bremerhaven, Germany

**Keywords:** Obesity, Endocrinology

## Abstract

**Background and significance:**

Obesity is a chronic disease, warranting long-term medical intervention. We evaluated effects of testosterone (T) therapy (Th) in men with T deficiency with normal weight, overweight and obesity on anthropometric and metabolic parameters, compared with untreated men.

**Methods:**

Hypogonadal men (*n* = 823) with total T ≤ 12.1 nmol/L (age: 60.6 ± 7.0 years) participated in an ongoing registry study. Among these men 474 (57.6%) were obese, 286 (34.8%) overweight and 63 (7.7%) had normal weight. T undecanoate injections were administered to 428 men and 395 remained untreated. Anthropometric and metabolic parameters were measured at least twice a year and changes adjusted for confounding factors to account for baseline differences between groups.

**Results:**

Long-term TTh in hypogonadal men, irrespective of weight at baseline, produced improvements in body weight, waist circumference (WC) and body mass index (BMI). Furthermore, TTh decreased fasting blood glucose and HbA_1c_ and improved lipid profiles. Gradual decreases in blood pressure (systolic and diastolic) and pulse pressure occurred in men treated with T in each group. Marked reductions in mortality and major cardiovascular events were recorded in men receiving TTh.

**Conclusions:**

Our findings demonstrate that TTh produces reductions in weight, WC, and BMI. There were 77 (19.5%) deaths in the untreated groups and 23 (5.4%) in the T-groups. Based on these findings we suggest that long-term TTh in overweight and obese hypogonadal men produces progressive and sustained clinically meaningful weight loss and that TTh may contribute to reductions in mortality and incident major adverse cardiovascular events.

## Introduction

Obesity is a serious public health concern, impacting healthcare systems worldwide [1–5], and warrants long-term medical intervention [4–7]. Approximately 78 million Americans are obese and 11 million are excessively obese making them eligible for surgical intervention [8]. Lung et al. [9] and Nyberg et al. [10] suggested that obesity was associated with the loss of disease-free years during middle and later adulthood. Herrick et al. reported that the age-adjusted percentage of adults aged ≥ 20 years trying to lose weight during the past 12 months, by sex, increased from 43.3% to 49.3%. This increase was seen among both men (35.5% to 42.2%) and women (51.2% to 56.3%) [11].

Several pathophysiological mechanisms including metabolic dysregulation and inability to regulate energy intake and expenditure, sedentary lifestyle and disruptions of endocrine function contribute to obesity. Current management strategies for the treatment of obesity include diets, exercise, behavioral lifestyle changes, pharmaco-therapeutic agents [12, 13], and bariatric surgery. Clearly, there is a critical need for new approaches to management of obesity [4].

Observational studies of patients with type 2 diabetes (T2DM) and severe obesity, who underwent bariatric surgery, reported improvements in the cardiovascular disease (CVD) risk factor profile, including metabolic syndrome, a lower risk of ischemic heart disease and mortality [14, 15], as well as prevention of new onset of diabetes as compared with other approaches, such as lifestyle and diet regimens [16]. Bariatric surgery, however, is not appropriate or available for all obese patients, and is not without risks and complications. Only carefully selected obese patients can be treated with bariatric surgery, and patients need to be followed-up closely and carefully [15].

Two recent comprehensive reviews suggested that TTh in obese men with TD may be considered as novel approach to treat obesity since it reduces fat mass and increases lean body mass [7, 17]. Recent studies have suggested that long-term TTh in men produces significant weight loss (WL) and decreases WC and BMI [18–20].

Dhindsa et al. [21] reported that T levels are lower in obese men, especially with a BMI > 40 kg/m² [22, 23], suggesting that obesity is associated with a high prevalence of hypogonadism. The inverse relation of free T with obesity is not restricted to middle-aged men but was also observed in boys and adolescents [24–26]. In addition to body composition, TTh improves mood, energy, vigor and overall quality of life [27–29]. The use of long-term TTh in obese hypogonadal men represents a novel, effective and safe intervention strategy in management of obesity in men with TD [18, 19, 30].

Recent recommendations by the American Association of Clinical Endocrinologists (AACE) [31] suggested that men with an increased WC or obesity should be assessed for hypogonadism; vice versa, all men with hypogonadism should be evaluated for the presence of overweight or obesity. Also, men with T2DM should be evaluated to exclude hypogonadism. Men with frank hypogonadism and obesity not seeking fertility should be considered for TTh, in addition to lifestyle intervention because TTh in these patients results in weight loss, decreased WC, and improvements in metabolic parameters (glucose, HbA_1c_, lipids, and blood pressure) [31].

We have previously reported on the effects of long-term TTh on anthropometric parameters in men with TD from our registry [18, 19]. In this report we compared long-term TTh in three groups of hypogonadal men, namely, normal weight, overweight and obese and evaluated the impact of TTh on anthropometric parameters, as compared with untreated controls in each group. Here we present data demonstrating that long-term TTh is a novel approach for treatment of overweight and obesity.

## Methods

A total of 823 men participating in an ongoing registry study in a urological office underwent physical and history examination as well as laboratory blood tests. Diagnosis of hypogonadism was made based on low T concentrations and clinical signs and symptoms associated with low T, as described by the European Association of Urology (EAU) guidelines. All men had total T ≤ 12.1 nmol/L and exhibited signs and symptoms of TD. Ethical guidelines by the German ‘Ärztekammer’ (German Medical Association) for observational studies in patients receiving standard treatment were followed. After receiving an explanation about the nature and the purpose of the study, all subjects provided written informed consent to be included in the registry and have their data analyzed.

It is important to note that some men opted against TTh following the advice of their family physician, or for financial reasons due to cost of medication, or for personal reasons due to negative perception of TTh. All men who opted against TTh were assigned to the control group and followed for the entire duration. Patients who opted to be treated with T, after consultation with their physician, were assigned to the treatment group. Therefore, allocation of patients to TTh group or untreated (control) group was based on the patients’ decision to accept or decline T treatment.

Among these 823 men, 474 (57.6%) were obese, 286 (34.8%) were overweight and 63 (7.7%) had normal weight. T undecanoate 1000 mg injections (TU) were administered every 12 weeks following an initial 6-week interval to 428 men. Three hundred and ninety-five remained untreated and served as controls. Measures of anthropometric and metabolic parameters were performed at least twice a year and changes adjusted for age, weight, WC, fasting glucose, blood pressure, lipid levels and the Aging Males’ Symptoms scale (AMS) to account for baseline differences between groups, as described previously [19, 32]. Myocardial infarctions (MIs), strokes or death were recorded based on hospital-issued reports.

Data were averaged across each year and obtained yearly data used to assess differences between the treated and untreated groups while adjusting for possible confounding. In adjusted multivariable analyses, changes from baseline were analyzed using a mixed model for repeated measures in terms of treatment, visit, and treatment-by-visit interaction as fixed factors and age, WC, weight, blood pressure, glucose, lipids, AMS as covariates. A random effect was included in the model for the intercept. Adjusted mean differences between treatment groups at each time point and across time within each treatment group were estimated using estimate statements in SAS PROCMIXED, Version 9.3 (2011) provided by SAS Institute Inc., Cary, North Carolina, USA. All statistical analyses were carried out as described [32].

## Results

### Baseline characteristics of normal weight, overweight and obese patients

Table 1 shows baseline characteristics, including the type of hypogonadism, comorbidities and concomitant medications in both treated and untreated groups.

**Table 1:** Baseline characteristics, comorbidities and concomitant medication at baseline in normal weight, overweight and obese men.

	Normal weight group treated (*n* = 26)	Normal weight group untreated control (*n* = 37)	*p* value between groups	Overweight group treated (*n* = 121)	Overweight group untreated control (*n* = 165)	*p* value between groups	Obese group treated (*n* = 281)	Obese group untreated control (*n* = 193)	*p* value between groups
Mean baseline age (years)	50.4 ± 8.4	63.8 ± 4.5	<0.0001	54.3 ± 7.7	64.0 ± 4.7	<0.0001	59.9 ± 6.0	63.5 ± 5.0	<0.0001
Mean follow-up (years)	7.4 ± 2.3	10.1 ± 1.0	NC	7.8 ± 3.1	9.6 ± 1.5	NC	8.7 ± 2.7	8.2 ± 2.6	NC
Median follow-up (years)	7	10	NC	8	10	NC	10	9	NC
Testosterone									
Total testosterone (nmol/L)	9.4 ± 1.1	9.5 ± 1.1	NS	9.3 ± 1.7	9.6 ± 1.1	NS	9.8 ± 1.5	9.8 ± 1.1	NS
Form of hypogonadism									
Klinefelter's syndrome (KS)	7 (26.9%)	0 (0.0%)	NC	22 (18.2%)	0 (0.0%)	NC	18 (6.4%)	0 (0.0%)	NC
Primary hypogonadism other than KS	1 (3.8%)	0 (0.0%)	NC	16 (13.2%)	0 (0.0%)	NC	11 (3.9%)	0 (0.0%)	NC
Functional hypogonadism	18 (69.2%)	37 (100.0%)	NC	83 (68.6%)	165 (100%)	NC	252 (89.7%)	193 (100%)	NC
Anthropometric parameters									
Weight (kg)	77.4 ± 4.3	77.0 ± 2.8	NS	87.1 ± 6.6	86.9 ± 5.6	NS	113.8 ± 11.5	105.0 ± 9.5	<0.0001
Waist circumference (cm)	95.4 ± 3.3	97.2 ± 3.0	<0.05	99 ± 4.6	105.1 ± 5.7	<0.0001	111.9 ± 10.8	119.1 ± 12.0	<0.0001
BMI (kg/m²)	24.1 ± 0.7	23.9 ± 0.7	NS	27.5 ± 1.9	27.8 ± 1.4	NS	36.4 ± 3.6	34.7 ± 5.3	<0.0001
Glycemic control									
HbA_1c_ (%)	5.8 ± 1.0	5.6 ± 1.0	NS	6.3 ± 1.6	5.8 ± 1.2	NS	8.0 ± 1.9	6.9 ± 1.4	<0.0001
Fasting glucose (mmol/L)	5.5 ± 0.6	5.5 ± 0.3	NS	5.7 ± 0.9	5.6 ± 0.4	NS	6.6 ± 1.4	6.0 ± 0.8	<0.0001
Blood pressure and hemodynamic parameters									
Systolic blood pressure (mmHg)	134.7 ± 9.4	136.7 ± 12.2	NS	140.8 ± 14.8	139.5 ± 11.4	NS	158.2 ± 15.5	145.2 ± 15.6	<0.0001
Diastolic blood pressure (mmHg)	78.0 ± 7.8	79.0 ± 7.2	NS	84.2 ± 9.7	79.7 ± 7.3	<0.0001	94.4 ± 11.3	83.5 ± 10.4	<0.0001
Heart rate (bpm)	75.7 ± 2.8	76.5 ± 4.6	NS	76.6 ± 3.3	76.7 ± 4.9	NS	78.3 ± 4.0	77.1 ± 4.7	<0.005
Pulse pressure (mmHg)	56.7 ± 5.2	57.6 ± 6.5	NS	56.6 ± 8.2	59.7 ± 6.5	<0.0005	63.8 ± 7.4	61.7 ± 8.7	<0.01
Lipids									
Total cholesterol (mmol/L)	6.9 ± 0.5	5.0 ± 0.6	<0.0001	7.2 ± 0.8	6.1 ± 1.0	<0.0001	8.2 ± 1.0	7.3 ± 1.1	<0.0001
HDL cholesterol (mmol/L)	0.8 ± 0.2	1.1 ± 0.2	<0.0005	1.0 ± 0.3	1.4 ± 0.5	<0.0001	1.1 ± 0.4	1.1 ± 0.5	NS
LDL cholesterol (mmol/L)	3.4 ± 0.6	2.1 ± 0.5	<0.0001	4.0 ± 0.8	3.0 ± 1.0	<0.0001	4.5 ± 0.8	4.1 ± 1.3	<0.0005
Triglyceride (mmol/L)	2.4 ± 0.3	2.3 ± 0.3	NS	2.8 ± 0.5	2.8 ± 0.4	NS	3.4 ± 0.6	3.2 ± 0.5	<0.005
Non-HDL cholesterol (mmol/L)	9.0 ± 2.3	4.9 ± 1.1	<0.0001	8.1 ± 2.7	5.0 ± 2.2	<0.0001	8.7 ± 3.4	7.9 ± 3.7	<0.05
Remnant cholesterol (mmol/L)	2.7 ± 0.8	1.8 ± 0.3	<0.0001	2.3 ± 0.80	1.7 ± 0.7	<0.0001	2.7 ± 0.9	2.1 ± 1.0	<0.0001
Quality of life									
AMS	46.4 ± 10.8	40.5 ± 6.0	<0.01	51.6 ± 9.3	40.4 ± 5.3	<0.0001	52.8 ± 10.1	40.2 ± 5.6	<0.0001
Prostate parameters									
Prostate volume (mL)	25.2 ± 9.5	33.2 ± 5.2	<0.0001	24.6 ± 8.4	35.0 ± 6.0	<0.0001	32.6 ± 10.2	35.6 ± 6.2	<0.0005
PSA (ng/mL)	1.4 ± 0.9	2.1 ± 1.4	<0.05	1.7 ± 1.1	2.4 ± 1.3	<0.0001	1.9 ± 0.9	2.6 ± 1.3	<0.0001
Erythropoiesis									
Hemoglobin (g/dL)	13.9 ± 0.8	14.7 ± 0.5	<0.0001	14.3 ± 0.7	14.7 ± 0.5	<0.0001	14.6 ± 0.6	14.7 ± 0.5	<0.01
Hematocrit (%)	42.9 ± 3.0	45.9 ± 1.1	<0.0001	43.3 ± 2.9	45.6 ± 1.3	<0.0001	44.3 ± 2.4	45.9 ± 1.4	<0.0001
Concomitant medication at baseline									
Statins	1 (3.8%)	0.0 (0%)	NS	18 (14.9%)	65 (39.4%)	<0.0001	159 (56.6%)	159 (82.4%)	<0.0001
Antidiabetic medication	2 (7.7%)	6 (16.2%)	NS	20 (16.5%)	43 (26.1%)	NS	143 (50.9%)	124 (64.2%)	<0.005
Antihypertensive medication	0 (0.0%)	7 (18.9%)	<0.05	25 (20.7%)	42 (25.5%)	NS	185 (65.8%)	93 (48.2%)	<0.0005
PDE5 inhibitors	11 (42.3%)	5 (13.5%)	<0.01	28 (23.1%)	32 (19.4%)	NS	58 (20.6%)	51 (26.4%)	NS
Comorbidities at baseline									
Type 2 diabetes	1 (3.8%)	6 (16.2%)	NS	17 (14.0%)	43 (26.1%)	<0.05	134 (47.7%)	123 (63.7%)	<0.001
Hypertension	17 (65.4%)	26 (70.3%)	NS	96 (79.4%)	121 (73.3%)	NS	275 (97.9%)	155 (80.3%)	<0.0001
Prior cardiovascular disease^a^	17 (65.4%)	26 (70.3%)	NS	12 (9.9%)	38 (23.0%)	<0.005	75 (26.7%)	76 (39.4%)	<0.005

Data are shown as means ± SD

^a^Cardiovacular disease was defined as prior myocardial infarction, stroke, or diagnosis of coronary artery disease

### Effects of long-term TTh on anthropometric parameters in hypogonadal men with normal weight, overweight or obesity at baseline

**Fig. 1:**
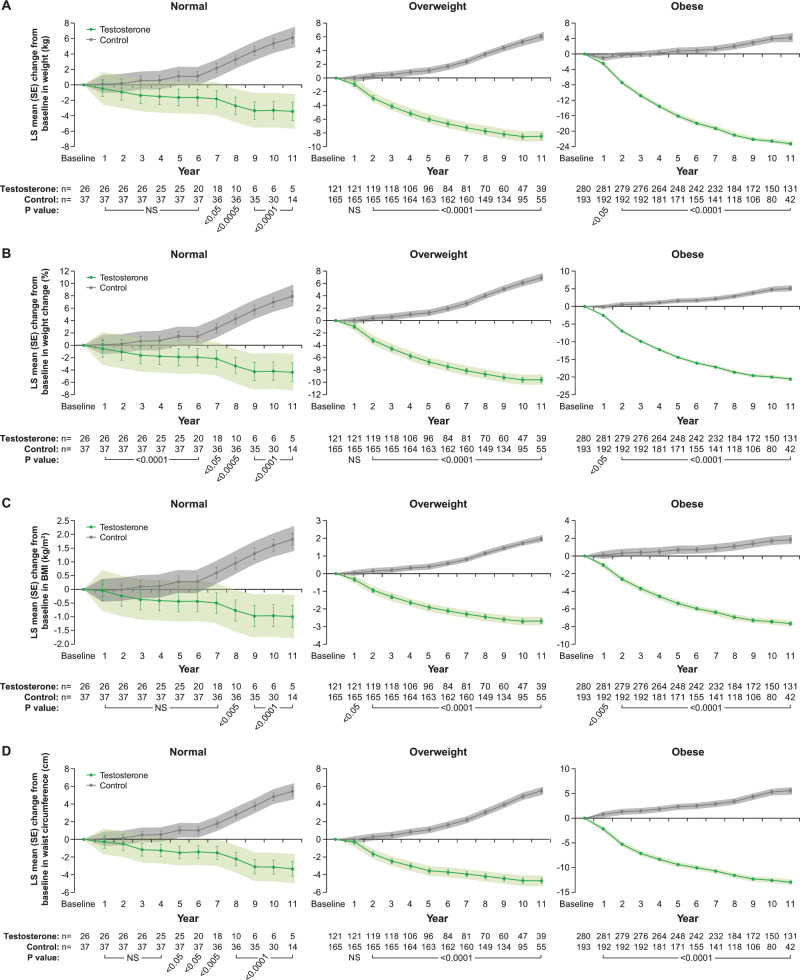
**a** Changes in weight (kg) in hypogonadal men with normal weight (left), overweight (middle) or obesity (right) treated with or without testosterone therapy. **b** Percent changes in weight in hypogonadal men with normal weight, overweight or obesity, at baseline, treated with or without testosterone therapy. **c** Changes in BMI (kg/m²) in hypogonadal men with normal weight, overweight or obesity at baseline treated with or without testosterone therapy. **d** Changes in waist circumference in hypogonadal men with normal weight, overweight or obese at baseline treated with or without testosterone therapy. Data are shown as least squares means ± standard errors. Shaded areas represent 95% confidence intervals. *P* values indicate statistical significance between groups for each year.

At 11 years, in 26 men with normal weight receiving TTh, weight decreased by 3.4 ± 1.2 kg (*p* < 0.005) and increased by 6.1 ± 0.7 kg (*p* < 0.0001) in 37 men with normal weight who remained untreated (Fig. 1a, left panel). Similarly, in 113 overweight men on TTh, weight decreased by 8.5 ± 0.4 kg and increased by 6.0 ± 0.3 kg (*p* < 0.0001 for both) in 167 untreated overweight men (Fig. 1a, middle panel). In 281 obese men on TTh, weight decreased by 23.2 ± 0.3 kg and increased by 4.2 ± 0.5 kg (*p* < 0.0001 for both) in 193 untreated obese men (Fig. 1a, right panel).

When % weight change was analyzed, men with normal weight on TTh lost 4.8 ± 1.5% (*p* < 0.005) while untreated men gained 8.0 ± 0.9% (*p* < 0.0001) (Fig. 1b, left panel). Men with overweight on TTh lost 9.6 ± 0.4% while untreated men gained 6.9 ± 0.3% (*p* < 0.0001 for both) (Fig. 1b, middle panel). Obese men on TTh lost 20.6 ± 0.3% while untreated obese men gained 5.1 ± 0.4% (*p* < 0.0001 for both) (Fig. 1b, right panel). The changes in weight with and without TTh in all three groups were reflected in corresponding changes in BMI (Fig. 1c).

In men with normal weight receiving TTh, WC decreased by 3.4 ± 0.8 cm and increased in untreated men by 5.5 ± 0.5 cm (*p* < 0.0001 for both) (Fig. 1d, left panel). In men with overweight on TTh, WC decreased by 4.7 ± 0.3 cm and increased by 5.5 ± 0.2 cm (*p* < 0.0001 for both) in untreated overweight men (Fig. 1d, middle panel). In obese men on TTh, WC decreased by 12.9 ± 0.2 cm and increased by 5.6 ± 0.4 cm (*p* < 0.0001 for both) in untreated obese men (Fig. 1d, right panel).

### Effects of long-term TTh on blood pressure and pulse pressure in men with testosterone deficiency (TD) and normal weight, overweight or obesity at baseline

**Fig. 2:**
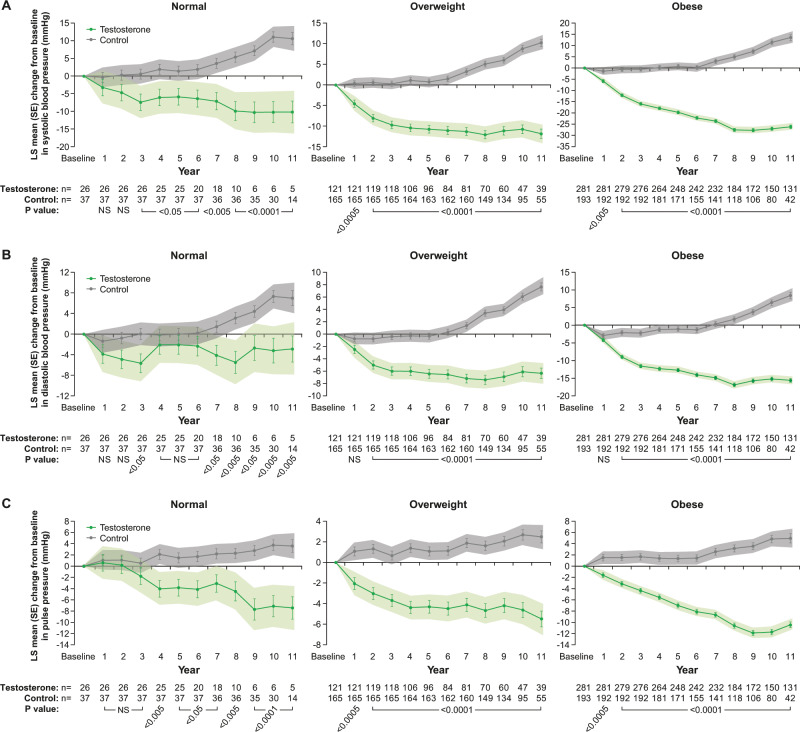
**a** Changes in systolic blood pressure (mmHg) in hypogonadal men with normal weight, overweight or obesity at baseline treated with or without testosterone therapy. **b** Changes in diastolic blood pressure (mmHg) in hypogonadal men with normal weight, overweight or obesity at baseline treated with or without testosterone therapy. **c** Changes in pulse pressure in hypogonadal men with normal weight, overweight or obesity at baseline treated with or without testosterone therapy. Data are shown as least squares means ± standard errors. Shaded areas represent 95% confidence intervals. *P* values indicate statistical significance between groups for each year.

In men with normal weight receiving TTh, systolic blood pressure (SBP) decreased by 10.2 ± 3.1 mmHg (*p* < 0.005) and increased by 10.6 ± 1.8 mmHg (*p* < 0.0001) in untreated men with normal weight (Fig. 2a, left panel). In men with overweight on TTh, SBP decreased by 11.8 ± 1.1 mmHg and increased by 10.2 ± 0.9 mmHg (*p* < 0.0001 for both) in untreated overweight men (Fig. 2a, middle panel). In obese men on TTh, SBP decreased by 26.1 ± 0.8 mmHg and increased by 13.5 ± 1.3 mmHg (*p* < 0.0001 for both) in untreated obese men (Fig. 2a, right panel).

In men with normal weight on TTh, diastolic blood pressure (DBP) decreased by 2.9 ± 2.5 mmHg (NS) and increased by 7.0 ± 1.5 mmHg (*p* < 0.0001) in untreated normal-weight men (Fig. 2b, left panel). In overweight men on TTh, DBP decreased by 6.3 ± 0.9 mmHg and increased by 7.6 ± 0.7 mmHg (*p* < 0.0001 for both) in untreated overweight men (Fig. 2b, middle panel). In obese men on TTh, DBP decreased by 15.6 ± 0.6 mmHg and increased by 8.4 ± 0.9 mmHg (*p* < 0.0001 for both) in untreated obese men (Fig. 2b, right panel).

In men with normal weight on TTh, pulse pressure (PP) decreased by 7.4 ± 2.0 mmHg (*p* < 0.0005) and increased by 3.6 ± 1.1 mmHg (*p* < 0.005) in untreated men (Fig. 2c, left panel). In overweight men on TTh, PP decreased by 5.5 ± 0.8 mmHg and increased by 2.5 ± 0.6 mmHg (*p* < 0.0001 for both) in untreated men (Fig. 2c, middle panel). In obese men on TTh, PP decreased by 10.5 ± 0.5 mmHg and increased by 5.0 ± 0.8 mmHg (*p* < 0.0001 for both) in untreated obese men (Fig. 2c, right panel).

### Effects of long-term TTh on lipid pattern in hypogonadal men with normal weight, overweight or obesity at baseline

**Fig. 3:**
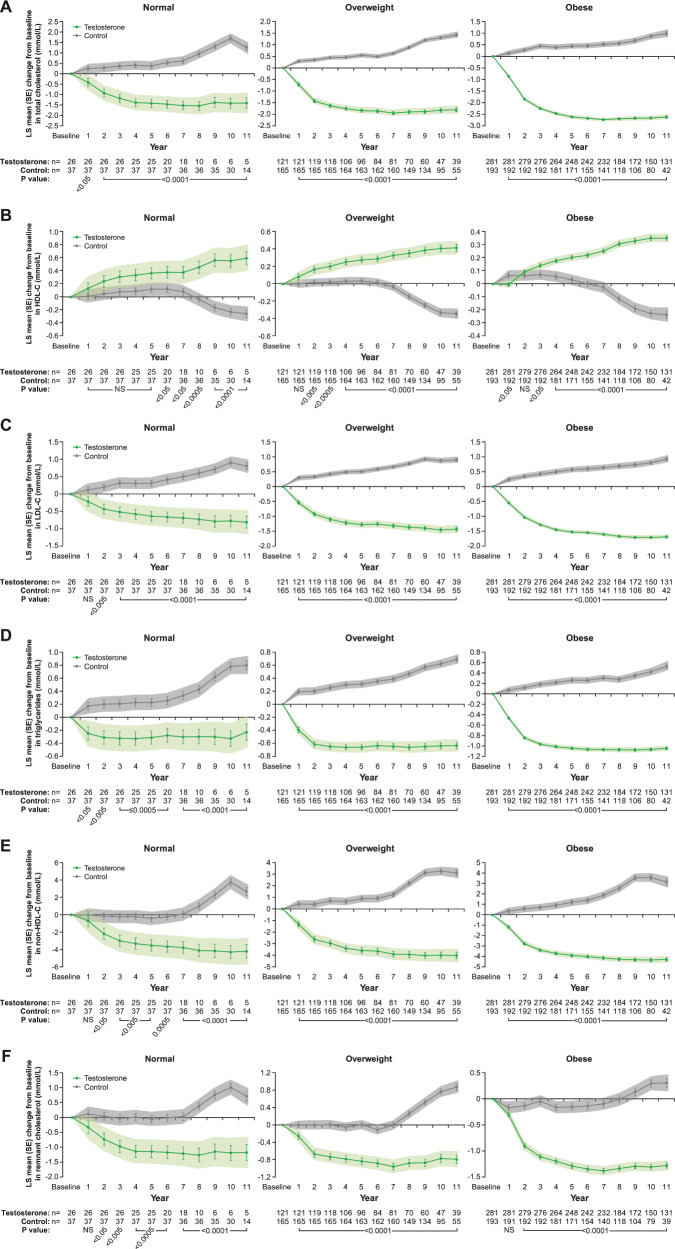
**a** Changes in total cholesterol (mmol/L) in hypogonadal men with normal weight (left), overweight (middle) or obesity (right) treated with or without testosterone therapy. **b** Changes in HDL (mmol/L) in hypogonadal men with normal weight (left), overweight (middle) or obesity (right) treated with or without testosterone therapy. **c** Changes in LDL (mmol/L) in hypogonadal men with normal weight (left), overweight (middle) or obesity (right) treated with or without testosterone therapy. **d** Changes in triglycerides (mmol/L) in hypogonadal men with normal weight (left), overweight (middle) or obesity (right) treated with or without testosterone therapy. **e** Changes in Non-HDL cholesterol (mmol/L) in hypogonadal men with normal weight (left), overweight (middle) or obesity (right) treated with or without testosterone therapy. **f** Changes in remnant cholesterol (mmol/L) in hypogonadal men with normal weight (left), overweight (middle) or obesity (right) treated with or without testosterone therapy. Data are shown as least squares means ± standard errors. Shaded areas represent 95% confidence intervals. *P* values indicate statistical significance between groups for each year.

In men with normal weight on TTh, total cholesterol (TC) decreased by 1.4 ± 0.2 mmol/L and increased by 1.3 ± 0.1 mmol/L (*p* < 0.0001 for both) in untreated men with normal weight (Fig. 3a, left panel). In overweight men on TTh, TC decreased by 1.8 ± 0.1 mmol/L and increased by 1.4 ± 0.1 mmol/L (*p* < 0.0001 for both) in untreated overweight men (Fig. 3a, middle panel). In obese men on TTh, TC decreased by 2.6 ± 0.0 mmol/L and it increased by 1.0 ± 0.1 mmol/L (*p* < 0.0001 for both) in untreated obese men (Fig. 3a, right panel).

In men with normal weight on TTh, HDL increased by 0.6 ± 0.1 mmol/L and decreased by 0.3 ± 0.1 mmol/L (*p* < 0.0001 for both) in untreated men (Fig. 3b, left panel). In men with overweight on TTh, HDL increased by 0.4 ± 0.0 mmol/L and decreased by 0.3 ± 0.0 mmol/L (*p* < 0.0001 for both) in untreated men (Fig. 3b, middle panel). In obese men on TTh, HDL increased by 0.4 ± 0.0 mmol/L and decreased by 0.2 ± 0.0 mmol/L (*p* < 0.0001 for both) in untreated men (Fig. 3b, right panel).

In men with normal weight on TTh, LDL decreased by 0.8 ± 0.2 mmol/L and increased by 0.8 ± 0.1 mmol/L (*p* < 0.0001 for both) in untreated men (Fig. 3c, left panel). In men with overweight on TTh, LDL decreased by 1.4 ± 0.1 mmol/L and increased by 0.9 ± 0.1 mmol/L (*p* < 0.0001 for both) in untreated men (Fig. 3c, middle panel). In obese men on TTh, LDL decreased by 1.7 ± 0.0 mmol/L and increased by 0.9 ± 0.1 mmol/L (*p* < 0.0001 for both) in untreated men (Fig. 3c, right panel).

In men with normal weight on TTh, triglyceride (TG) decreased by 0.2 ± 0.1 mmol/L (NS) and increased by 0.8 ± 0.1 mmol/L (*p* < 0.0001) in untreated men (Fig. 3d, left panel). In men with overweight on TTh, TG decreased by 0.6 ± 0.0 mmol/L and increased by 0.7 ± 0.0 mmol/L (*p* < 0.0001 for both) in untreated men (Fig. 3d, middle panel). In obese men on TTh, TG decreased by 1.0 ± 0.0 mmol/L and increased by 0.5 ± 0.0 mmol/L (*p* < 0.0001 for both) in untreated men (Fig. 3d, right panel).

In men with normal weight on TTh, Non-HDL decreased by 4.2 ± 0.8 mmol/L and increased by 2.7 ± 0.4 mmol/L (*p* < 0.0001 for both) in untreated men (Fig. 3e, left panel). In men with overweight on TTh, Non-HDL decreased by 4.0 ± 0.3 mmol/L and increased by 3.1 ± 0.2 mmol/L (*p* < 0.0001 for both) in untreated men (Fig. 3e, middle panel). In obese men on TTh, Non-HDL decreased by 4.3 ± 0.2 mmol/L and increased by 3.2 ± 0.3 mmol/L (*p* < 0.0001 for both) in untreated men (Fig. 3e, right panel).

In men with normal weight on TTh, remnant cholesterol decreased by 1.2 ± 0.3 mmol/L and increased by 0.7 ± 0.2 mmol/L (*p* < 0.0001 for both) in untreated men (Fig. 3f, left panel). In men with overweight on TTh, remnant cholesterol decreased by 0.8 ± 0.1 mmol/L and increased by 0.9 ± 0.1 mmol/L (*p* < 0.0001 for both) in untreated men (Fig. 3f, middle panel). In obese men on TTh, remnant cholesterol decreased by 1.3 ± 0.1 mmol/L (*p* < 0.0001) and increased by 0.3 ± 0.1 mmol/L (*p* < 0.001) in untreated men (Fig. 3f, right panel).

### Effects of long-term TTh on serum T levels in hypogonadal men with normal weight, overweight or obesity at baseline

In men with normal weight on TTh, T increased by 8.9 ± 0.5 nmol/L (*p* < 0.0001) and decreased by 1.0 ± 0.3 nmol/L (*p* < 0.005) in untreated men (Supplementary Fig. 1, left panel). In men with overweight on TTh, T increased by 8.5 ± 0.2 nmol/L and decreased by 1.6 ± 0.2 nmol/L (*p* < 0.0001 for both) in untreated men (Supplementary Fig. 1, middle panel). In obese with obesity on TTh, T increased by 7.9 ± 0.1 nmol/L and decreased by 2.4 ± 0.3 nmol/L (*p* < 0.0001 for both) in untreated men (Supplementary Fig. 1, right panel).

### Effects of long-term TTh on glycemic control in hypogonadal men with normal weight, overweight or obesity at baseline

In men with normal weight on TTh, fasting plasma glucose (FPG) decreased by 0.3 ± 0.2 mmol/L (NS) and increased by 0.8 ± 0.1 mmol/L (*p* < 0.0001) in untreated men (Supplementary Fig. 2A, left panel). In men with overweight on TTh, FPG decreased by 0.3 ± 0.2 mmol/L (NS) and increased by 0.8 ± 0.2 mmol/L (*p* < 0.0001) in untreated men (Supplementary Fig. 2A, middle panel). In obese men on TTh, FPG decreased by 1.0 ± 0.1 mmol/L and increased by 1.8 ± 0.1 mmol/L (*p* < 0.0001 for both) in untreated men (Supplementary Fig. 2A, right panel).

In men with normal weight on TTh, HbA_1c_ decreased by 0.2 ± 0.6% (NS) and increased by 1.7 ± 0.2% (*p* < 0.0001) in untreated men (Supplementary Fig. 2B, left panel). In men with overweight on TTh, HbA_1c_ decreased by 1.5 ± 0.2% and increased by 1.8 ± 0.1% (*p* < 0.0001 for both) in untreated men (Supplementary Fig. 2B, middle panel). In obese men on TTh, HbA_1c_ decreased by 2.2 ± 0.2% and increased by 2.8 ± 0.3% (*p* < 0.0001 for both) in untreated men (Supplementary Fig. 2B, right panel).

### Major adverse events

As shown in Table 3, in men with normal weight receiving TTh, there was one death, and there was no occurrence of nonfatal MIs and strokes. In untreated men with normal weight, there were three deaths, four nonfatal MIs and six nonfatal strokes. In overweight men receiving TTh, there were four deaths, no nonfatal MIs and strokes. In untreated overweight men, there were 14 deaths, 21 nonfatal MIs and 11 nonfatal strokes. In obese men receiving TTh, there were 18 deaths, no nonfatal MIs and strokes. In untreated obese men, there were 60 deaths, 49 nonfatal MIs and 50 nonfatal strokes. In overweight men, incidence of MIs and strokes in untreated controls were statistically significantly higher compared with the T-treated men (*p* < 0.0001 and *p* < 0.005, respectively). In obese men, incidence of deaths, MIs and strokes were statistically significantly higher compared with T-treated men (*p* < 0.0001 for all three).

There was one prostate cancer in the untreated group (NS) with normal weight. Prostate cancer was diagnosed in 5 (4.1%) overweight men treated and 15 (9.1%) untreated men (NS). Prostate cancer was diagnosed in 7 (2.5%) obese men who were T-treated and 29 men (15.0%) who remained untreated (*p* < 0.0001). As expected, there were slight increases in PSA in both treated and untreated groups. Changes in PSA are shown in detail in Supplementary Table 2. As expected, there were slight increases in hemoglobin and hematocrit in the T-treated groups, but levels remained within the physiological reference ranges. We have not encountered a single case of deep vein thrombosis. It is worth noting that we have not encountered any patient with hematocrit greater than 54% in two consecutive measurements and therefore no actions were taken.

## Discussion

Our main findings demonstrate that long-term TTh in hypogonadal men, irrespective of baseline weight, resulted in significant improvements in body weight, WC and BMI. The most significant observation was that weight loss was progressive and sustained in all three groups receiving TTh. In contrast, weight gain was observed in all untreated groups. When results were compared between groups, men with normal weight approached 5% weight loss, men with overweight 10% and men with obesity 20% weight loss; these values represent significant weight loss that was previously unmatched by any other therapeutic intervention, except for bariatric surgery. Similar findings were also recorded for WC. We should point out that the mean baseline age (years) was lower in the T-treated groups, as compared with the untreated groups. This may be attributed, in part, to men with Klinefelter’s syndrome (*n* = 47) comprising 11.0% of the T-groups or men who had undergone orchiectomy following testicular cancer who were considerably younger and in whom TTh was mandatory.

Our findings suggest that the estimated differences for anthropometric parameters, at 11 years, between T-treated and control subjects, after adjustments for confounding factors, were significant (Table 2). In hypogonadal men with normal weight, overweight and obesity with or without TTh, estimated adjusted differences in weight between groups were 9.5, 14.5, and 27.4 kg, respectively. Accordingly, changes in BMI were 2.8, 4.7, and 9.5 kg/m², respectively. Estimated adjusted differences in WC between groups were 8.8, 10.2, and 18.5 cm, respectively. We also noted significant changes between groups in glycemic control, blood pressure and PP, lipid profiles and quality of life (Table 2). The most relevant observation noted in this study is that men who were obese had the greatest benefit from TTh, in all assessed parameters. It is worth noting that the magnitude of changes with TTh was greater in men with the least favorable conditions in terms of cardiometabolic risk profile at baseline.

**Table 2:** Estimated differences at 11 years between treated and control patients, adjusted for baseline age, BMI, fasting glucose, lipids, blood pressure, and quality of life (assessed by AMS).

	Normal weight group treated (*n* = 26) vs control (*n* = 37)				Overweight group treated (*n* = 113) vs control (*n* = 167)				Obese group treated (*n* = 281) vs control (*n* = 193)			
	Estimated adjusted difference between groups	95% confidence interval		*p* value between groups	Estimated adjusted difference between groups	95% confidence interval		*p* value between groups	Estimated adjusted difference between groups	95% confidence interval		*p* value between groups
		Lower	Upper			Lower	Upper			Lower	Upper	
Testosterone												
Total testosterone (nmol/L)	9.5	8.1	10.9	<0.0001	10.1	9.5	10.7	<0.0001	10.3	9.7	11.0	<0.0001
Anthropometric parameters												
Weight (kg)	−9.5	−12.8	−6.3	<0.0001	−14.5	−15.6	−13.4	<0.0001	−27.4	−28.8	−26.0	<0.0001
Weight change (%)	−12.3	−16.6	−8.1	<0.0001	−16.5	−17.8	−15.3	<0.0001	−25.6	−26.8	−24.5	<0.0001
BMI (kg/m²)	−2.8	−4.0	−1.7	<0.0001	−4.7	−5.0	−4.3	<0.0001	−9.5	−10.3	−8.8	<0.0001
Waist circumference (cm)	−8.8	−11.1	−6.5	<0.0001	−10.2	−11.1	−9.3	<0.0001	−18.5	−19.5	−17.5	<0.0001
Blood pressure and hemodynamic parameters												
Systolic blood pressure (mmHg)	−20.8	−28.8	−12.7	<0.0001	−22.0	−25.2	−18.9	<0.0001	−39.6	−42.9	−36.4	<0.0001
Diastolic blood pressure (mmHg)	−9.9	−16.4	−3.3	<0.005	−14.0	−16.4	−11.6	<0.0001	−24.0	−26.4	−21.7	<0.0001
Heart rate (bpm)	−3.2	−7.9	1.6	NS	−2.9	−4.4	−1.4	<0.0005	−3.8	−5.0	−2.5	<0.0001
Pulse pressure (mmHg)	−11.0	−16.2	−5.8	<0.0001	−8.0	−10.1	−5.6	<0.0001	−15.4	−17.5	−13.3	<0.0001
Lipids												
Total cholesterol (mmol/L)	−2.7	−3.3	−2.0	<0.0001	−3.2	−3.5	−3.0	<0.0001	−3.6	−3.8	−3.4	<0.0001
HDL cholesterol (mmol/L)	0.9	0.6	1.1	<0.0001	0.8	0.6	0.9	<0.0001	0.6	0.5	0.7	<0.0001
LDL cholesterol (mmol/L)	−1.6	−2.1	−1.1	<0.0001	−2.3	−2.5	−2.1	<0.0001	−2.6	−2.8	−2.5	<0.0001
Triglyceride (mmol/L)	−1.0	−1.4	−0.7	<0.0001	−1.3	−1.5	−1.2	<0.0001	−1.6	−1.7	−1.5	<0.0001
Non-HDL cholesterol (mmol/L)	−6.9	−9.0	−4.8	<0.0001	−7.1	−8.0	−6.3	<0.0001	−7.5	−8.1	−6.8	<0.0001
Remnant cholesterol (mmol/L)	−1.9	−2.6	−1.2	<0.0001	−1.7	−1.9	−1.4	<0.0001	−1.6	−1.8	−1.4	<0.0001
Glycemic control												
HbA_1c_ (%)	−1.9	−3.2	−0.5	<0.01	−3.4	−3.9	−2.9	<0.0001	−5.1	−5.9	−4.3	<0.0001
Fasting glucose (mmol/L)	−1.0	−1.6	−0.5	<0.0005	−1.2	−1.8	−0.5	<0.0005	−2.8	−3.1	−2.6	<0.0001
Quality of life												
AMS	−47.9	−52.1	−43.6	<0.0001	−43.8	−45.8	−41.9	<0.0001	−50.3	−51.9	−48.6	<0.0001
Prostate parameters												
Prostate volume (mL)	−0.7	−2.8	1.3	NS	−0.3	−0.5	1.1	NS	−0.4	−1.3	−0.5	NS
PSA (ng/mL)	−1.7	−2.7	−0.7	<0.001	−0.7	−1.2	−0.2	<0.01	−0.2	−0.7	0.2	NS
Erythropoiesis												
Hemoglobin (g/dL)	1.0	0.6	1.5	<0.0001	0.4	0.3	0.6	<0.0001	0.6	0.4	0.7	<0.0001
Hematocrit (%)	4.7	2.3	7.1	<0.0005	2.4	1.6	3.1	<0.0001	2.0	1.3	2.6	<0.0001

*NS* non-significant

In all three groups, TTh decreased fasting blood glucose, while there was an increase in the untreated men in each group. Similarly, irrespective of baseline weight, TTh resulted in progressive decline of HbA_1c_ while there was an increase in the untreated men. We also noted that, irrespective of weight at baseline, lipid profiles were improved in men treated with T but not in the untreated men. This is reflected in increased HDL levels, decreased TC, LDL and TG levels, and marked reductions in non-HDL and remnant cholesterol. Since it is believed that increased TG, reduced HDL, increased LDL, and increased remnant cholesterol levels (primarily VLDL, see below), represent markers of atherogenic or ‘adiposopathic” dyslipidemia [33], one would expect that significant improvements in cardiometabolic parameters, such as improved lipid profile, glycemic control, blood pressure and PP in response to TTh in overweight and obese men would reduce deaths and other major cardiovascular events. As noted in Table 3, indeed TTh in overweight and obese men resulted in marked reduction in major cardiovascular adverse events. In contrast, untreated men experienced increased incidence of deaths and major adverse events including prostate cancer, confirming previous studies [32, 34].

**Table 3:** Rates of patients with adverse events by weight groups (normal weight, overweight, obese) and all patients. MACE is defined as death or nonfatal myocardial infarction or nonfatal stroke.

	Normal weight group treated (*n* = 26)	Normal weight group untreated control (*n* = 37)	*p* value between groups	Overweight group treated (*n* = 121)	Overweight group untreated control (*n* = 165)	*p* value between groups	Obese group treated (*n* = 281)	Obese group untreated control (*n* = 193)	*p* value between groups	ALL Treated (*n* = 428)	ALL Untreated control (*n* = 395)	*p* value between groups
Deaths	1 (3.8%)	3 (8.1%)	NS	4 (3.3%)	14 (8.5%)	NS	18 (6.4%)	60 (31.1%)	<0.0001	23 (5.4%)	77 (19.5%)	<0.0001
Nonfatal myocardial infarctions	0 (0%)	4 (10.8%)	NS	0 (0%)	21 (12.7%)	<0.0001	0 (0%)	49 (25.4%)	<0.0001	0 (0%)	74 (18.7%)	<0.0001
Nonfatal strokes	0 (0%)	6 (16.2%)	<0.05	0 (0%)	11 (6.7%)	<0.005	0 (0%)	50 (25.9%)	<0.0001	0 (0%)	67 (17.0%)	<0.0001
Prostate cancer	0 (0%)	1 (2.7%)	NS	5 (4.1%)	15 (9.1%)	NS	7 (2.5%)	29 (15.0%)	<0.0001	12 (2.8%)	45 (11.4%)	<0.05
MACE	1 (3.9%)	8 (21.6%)	NS	4 (3.3%)	38 (23.0%)	<0.0001	18 (6.4%)	98 (50.8)	<0.0001	23 (5.4%)	144 (36.5%)	<0.0001

*NS* non-significant

Our findings showed meaningful decreases in SBP and DBP in men treated with T in each group but significant increases in blood pressure in untreated men in each group, irrespective of weight at baseline. The decrease in blood pressure in the T-treated men was maintained over the entire course of TTh. A potential link between hypogonadism and risk of hypertension and the improvement in blood pressure with TTh was previously discussed [35, 36]. Hypogonadal men treated with T exhibited reduced blood pressure [37, 38]. SBP appears to be inversely associated with T levels, thus, it is possible that hypogonadism, via a host of biochemical and physiological mechanisms, contributes to higher blood pressure [37–39].

PP (the difference between systolic and diastolic pressure) is an independent marker of arterial stiffness and cardiovascular risk. In this study, we recorded the changes in PP in the T-groups and compared these measures with the untreated groups. Our findings showed meaningful decreases in PP in men treated with T but significant increases in PP in untreated men, irrespective of baseline weight. The decrease in PP in the T-treated men was maintained over the entire course of TTh. Because PP is a marker of vascular stiffness, any reduction in this parameter is viewed favorable in reducing CVD risk [35, 40, 41].

Our findings of TTh in obese men are worthy of further discussion. Accumulating evidence indicates that androgens are critical modulators of body fat distribution [20]. Data from observational studies show that reduced total T is frequently observed in men with abdominal and/or visceral obesity and in men with metabolic syndrome, and TTh decreases WC and BMI. Obesity is a chronic disease, warranting medical intervention and long-term Th. It is deemed critical that new therapeutic modalities are developed for the management of obesity and to augment behavioral lifestyle changes. Traish [30] and Traish and Zitzmann [7] advanced a framework suggesting that obesity directly impacts T levels and reduced T levels contribute to increased adiposity, setting a vicious cycle. This framework further suggests that a complex relationship between T and obesity exists, and this contributes to the hurdles of understanding the biochemical and pathophysiological mechanisms that underlie the obesity pathology. This framework advances the notion that TTh in obese hypogonadal men is a potential novel approach to treatment of obesity since it reduces fat mass and increases lean body mass [7, 30].

In a recent meta-analysis, Skinner et al. [42] demonstrated that TTh with intramuscular T preparations was associated with a 5.7% increase in lean body mass and 10–13% increases in total body strength, leg strength, and arm strength, and that the effect sizes were larger and per cent changes 3–5 times greater for intramuscular T preparations than for transdermal preparations vs. respective placebos, for all outcomes [42]. In the present study, patient adherence to TTh was 100 per cent as all injections were administered and documented in the urology office. Improvements in mood, energy, vigor and overall quality of life may have further contributed to achieving weight loss. The significance of long-term TTh in obese men with TD represents a novel effective and safe intervention strategy in management of obesity in men with TD [30, 32].

AACE indicated in their guidelines that all men who have an increased WC or who have obesity should be assessed for hypogonadism by history and physical examination and be offered TTh if indicated; all hypogonadal patients should be evaluated for the presence of overweight and obesity [31]. In an analysis of more than 120,000 adults free of cardiometabolic diseases at study entry, Kivimäki et al. [43] reported that overweight was associated with increased risk of developing cardiometabolic multimorbidity. Moreover, in patients with severe obesity, the risk is ten times greater than in subjects with normal weight. This association of overweight and obesity with cardiometabolic multimorbidity remained strong even after accounting for lifestyle behavior. Obese men have a 1.6 times increased risk of coronary heart disease [44] and a 1.6–1.8 times increased risk of stroke and admittance to hospital for any CVD [45, 46] when compared with men of a healthy weight. Severe obesity was linked to substantially greater disease risk, the odds ratios being almost 19-fold for diabetes alone and 30-fold for diabetes followed by vascular disease. Obesity increases the risk of dyslipidemia and systemic inflammation, which could be common pathways to the development of diabetes and vascular disease [47, 48].

### Study limitations

The nature of this observational registry study does not permit randomization and therefore allocation of patients to treatment group and untreated (control) group was made based on patients’ decisions to accept or decline T treatment. For this reason, potential bias may exist in the analysis of the results. However, we believe that the long follow-up duration allows reasonable assessment in the differences of various parameters between groups.

## Summary

Our findings demonstrate that in subpopulations of men in different weight categories at baseline, long-term TTh produced significant weight loss while untreated men in each subgroup (controls) had increased weight, WC, and BMI. We wish to point out that, in this study, the majority of hypogonadal men were obese. There were 77 deaths in the untreated groups and 23 deaths in the T-groups. No patient dropped out. Medication adherence to T was 100 per cent as all injections were administered in the doctor’s office and documented. We therefore suggest that adequate long-term TTh in hypogonadal overweight and obese men produces progressive and sustained, clinically meaningful weight loss and this Th may reduce mortality and incidence of major adverse cardiovascular events.

## Supplementary information

Supplementary Table 1

Supplementary Table 2

Supplementary Figure 1

Supplementary Figure 2
